# Is the Genetic Code Optimized for Resource Conservation?

**DOI:** 10.1093/molbev/msab239

**Published:** 2021-08-12

**Authors:** Haiqing Xu, Jianzhi Zhang

**Affiliations:** Department of Ecology and Evolutionary Biology, University of Michigan, Ann Arbor, MI, USA

**Keywords:** evolution, mutation, nitrogen, carbon, second-order selection

## Abstract

The causes and consequences of the nonrandom structure of the standard genetic code (SGC) have been of long-standing interest. A recent study reported that mutations in present-day protein-coding sequences are less likely to increase proteomic nitrogen and carbon uses under the SGC than under random genetic codes, concluding that the SGC has been selectively optimized for resource conservation. If true, this finding might offer important information on the environment in which the SGC and some of the earliest life forms evolved. However, we here show that the hypothesis of optimization of a genetic code for resource conservation is theoretically untenable. We discover that the aforementioned study estimated the expected mutational effect by inappropriately excluding mutations lowering resource consumptions and including mutations involving stop codons. After remedying these problems, we find no evidence that the SGC is optimized for nitrogen or carbon conservation.

##  

Because the atomic constituents vary among different nucleotides and amino acids, environmental nutrients can shape the nucleotide and amino acid compositions of a species through resource-driven selection (Elser et al. 2006, 2011; Grzymski and Dussaq 2012; Mende et al. 2017; Berube et al. 2019). For example, in the bacterium *Escherichia coli* and yeast *Saccharomyces cerevisiae*, the amino acid composition of the proteins in sulfur and carbon assimilation pathways is such that sulfur and carbon atoms are underrepresented relative to those in other proteins (Baudouin-Cornu et al. 2001). In the same vein, probably because every guanine–cytosine (GC) nucleotide pair uses eight nitrogen atoms whereas every adenine–thymine (AT) pair uses only seven nitrogen atoms, the genomic GC content is higher in nitrogen-fixing bacteria than in nonfixing members of the same genus (McEwan et al. 1998). Similarly, bacteria living in the deep sea, where the environmental nitrogen is abundant, have a higher GC content in their genomes and a higher nitrogen content in their proteomes when compared with surface-dwelling bacteria (Mende et al. 2017). In a recent study, Shenhav and Zeevi (2020) extended the analysis of the impact of environmental nutrients to the evolution of the genetic code. They reported that point mutations in protein-coding sequences are less likely to increase proteomic nitrogen and carbon uses under the standard genetic code (SGC) than under random genetic codes (RGCs), suggesting that the SGC has been optimized for resource conservation. This is reminiscent of the classic finding that coding mutations are more likely to conserve the physicochemical properties of the encoded amino acids under the SGC than under RGCs (Haig and Hurst 1991; Freeland and Hurst 1998; Archetti 2004; Goodarzi et al. 2004). If Shenhav and Zeevi’s conclusion is correct, it provides important information about the environment in which the SGC and some of the earliest life forms evolved. However, we find that the hypothesis of selective optimizion of the genetic code for resouce conservation is untenable because the optimzation requires a second-order selection that would be in the opposite direction of a much stronger first-order selection. Indeed, we show that Shenhav and Zeevi’s results are attributable to two problemetic assumptions in calculating the expected mutational cost.

### Contrasting First- and Second-Order Selections for Resource Conservation

Let us consider a hypothetical organism with only two codons, A and B. Under the wild-type genetic code, A encodes amino acid L that uses a low amount of a particular environmental resource, whereas B encodes amino acid H that uses a high amount of the resource (fig. 1). Let us assume that A and B often play similar functional roles in proteins. If the organism lives in an environment where the resource is limited, selection for resource conservation will lead to a higher frequency of A than B in the genome, for example, 80% of A and 20% of B. If all codons have the same probability of mutation, 80% of mutations will result in L-to-H changes whereas 20% result in H-to-L changes, causing an average mutation to increase the proteomic resource consumption (fig. 1). Now imagine a code-table-altering mutation that makes A code for H and B code for L. After the occurrence of this mutation, 80% of future mutations will result in H-to-L changes whereas 20% result in L-to-H changes, causing an average future mutation to decrease the proteomic resource consumption (fig. 1). Although this code-table-altering mutation is beneficial and favored by second-order selection for resource conservation when future mutations are considered, it is deleterious on arrival because it immediately increases the proteomic resource consumption given that 80% of the codons now code for the more costly amino acid (fig. 1). Therefore, the first-order selection for resource conservation will triumph over the second-order selection and prevent the fixation of the code-table-altering mutation. The principle illustrated by this toy example applies to any genetic code and organism, meaning that the hypothesis of optimization of a genetic code for resource conservation is not theoretically tenable.

**Fig. 1:**
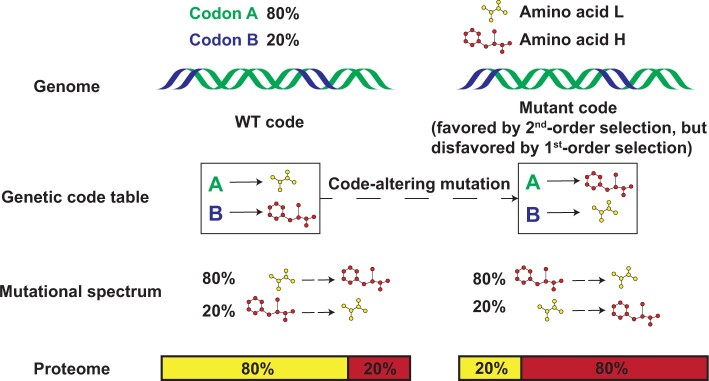
Schematics contrasting first- and second-order selections for resource conservation. A hypothetical organism living in a resource-limited environment has two codons; 80% of its codons are A and 20% are B. Under the wild-type (WT) genetic code, A encodes amino acid L that has a low cost of resource, whereas B encodes amino acid H that has a high cost of resource. Under the mutant code, A encodes H, whereas B encodes L. The code-table-altering mutation immediately increases the proteomic resource consumption but will lower the cost of future mutations, so the code-table-altering mutation is favored by the second-order selection but disfavored (to a greater extent) by the first-order selection for resource conservation. Codon-amino acid relationships are indicated by solid arrows, whereas mutations are indicated by broken arrows.

### Correcting ERMC Calculation Alters the Purported Resource Conservation of the SGC

To understand why Shenhav and Zeevi’s empirical results contradict the above theoretical conclusion, we examined how they calculated the expected random mutation cost (ERMC) in proteomic nitrogen/carbon usage under each code table. Surprisingly, they considered only the increase (positive cost) but not the decrease (negative cost) of the proteomic nitrogen/carbon content caused by mutations. In a nitrogen/carbon-limited environment, if mutations increasing the proteomic nitrogen/carbon content are deleterious, those lowering the content would be beneficial. Hence, the net expected random mutation cost (nERMC) should be the sum of the positive and negative costs of mutations. Additionally, Shenhav and Zeevi included in their ERMC calculation mutations that involve stop codons and treated the proteomic atom usage as 0 at stop codons. There are two types of point mutations involving stop codons. The first type converts a sense codon to a stop codon, causing premature termination of protein synthesis, whereas the second type converts a stop codon to a sense codon, leading to an extension of the protein sequence. In both cases, the mutations affect not only the proteomic atom usage at the mutated codon but also that due to the change in protein length, the latter being much greater than the former. Shenhav and Zeevi’s treatment of mutations involving stop codons is thus inappropriate. Considering that mutations involving stop codons are incomparable with missense mutations in their impacts on the resource consumption, we included only missense mutations in nERMC computation, which is also consistent with the general practice in the field (Haig and Hurst 1991; Freeland and Hurst 1998; Geyer and Madany Mamlouk 2018; Xu and Zhang 2021).

Shenhav and Zeevi (2020) reported that the “square” arrangement in the SGC, where nitrogen-rich amino acids are concentrated in one section of the code table instead of being spread over the entire table, reduces its ERMC. In fact, the “square” arrangement causes mutations to be less likely to increase as well as reduce the proteomic nitrogen usage. Specifically, if a mutation from codon *i* to *j* increases the nitrogen usage, this effect is completely offset by a reverse mutation from *j* to *i*. Let *μ*_ij_, the mutation rate from *i* to *j*, be the probability that a codon *i* is mutated to *j* in a unit time. When *μ*_ij_ equals *μ*_ji_ for all codon pairs—assumed in Shenhav and Zeevi (2020) and here—and when all codons are equally frequent, the mutational cost measured by nERMC is zero, because the expected numbers of forward and backward mutations between any two codons are equal. This result holds regardless of the structure of the code table or the transition/transversion mutation rate ratio (*κ*), a factor considered by Shenhav and Zeevi. In other words, under the above condition, the SGC is equally optimized as RGCs in nutrient conservation. Further, even when the above condition is not met but the number of mutations from any codon *i* to any codon *j* equals the number of mutations from *j* to *i*, the SGC is equally optimized as RGCs in nutrient conservation.

Under unequal codon frequencies (but equal mutation rates), nERMC varies among different code tables, with an expectation of zero across all RGCs. Regarding the SGC, if the frequencies of codons in a genome for nitrogen/carbon-rich amino acids are lower than those for nitrogen/carbon-poor amino acids, for example, as a result of the resource-driven selection aforementioned, mutations will tend to raise the proteomic nitrogen/carbon content, yielding a positive nERMC or a “less optimized” SGC than RGCs in nutrient conservation.

With the above consideration in mind, we turned to empirical data. For each of the 39 diverse species examined by Shenhav and Zeevi, we computed Pearson’s correlation across the 61 sense codons between the frequency of a codon (in the genome) and the number of nitrogen or carbon atoms in the amino acid encoded by the codon. For nitrogen, the correlation is negative in every species (fig. 2*A*), confirming the avoidance of codons encoding nitrogen-rich amino acids in these species (Grzymski and Dussaq 2012). For carbon, however, both positive and negative correlations are observed depending on the species concerned (fig. 2*A*). Because of the among-gene variation in expression level, we further computed codon frequencies in the transcriptome instead of the genome, and observed similar results (supplementary fig. S1*A*, Supplementary Material online) from the analysis of three bacterial and three unicellular eukaryotic species with available transcriptomic data (supplementary data S1, Supplementary Material online). We thus predict that, compared with RGCs, the SGC will not look optimized in nitrogen conservation but may look optimized for carbon conservation in those few species with strong codon preferences for carbon-rich amino acids.

**Fig. 2:**
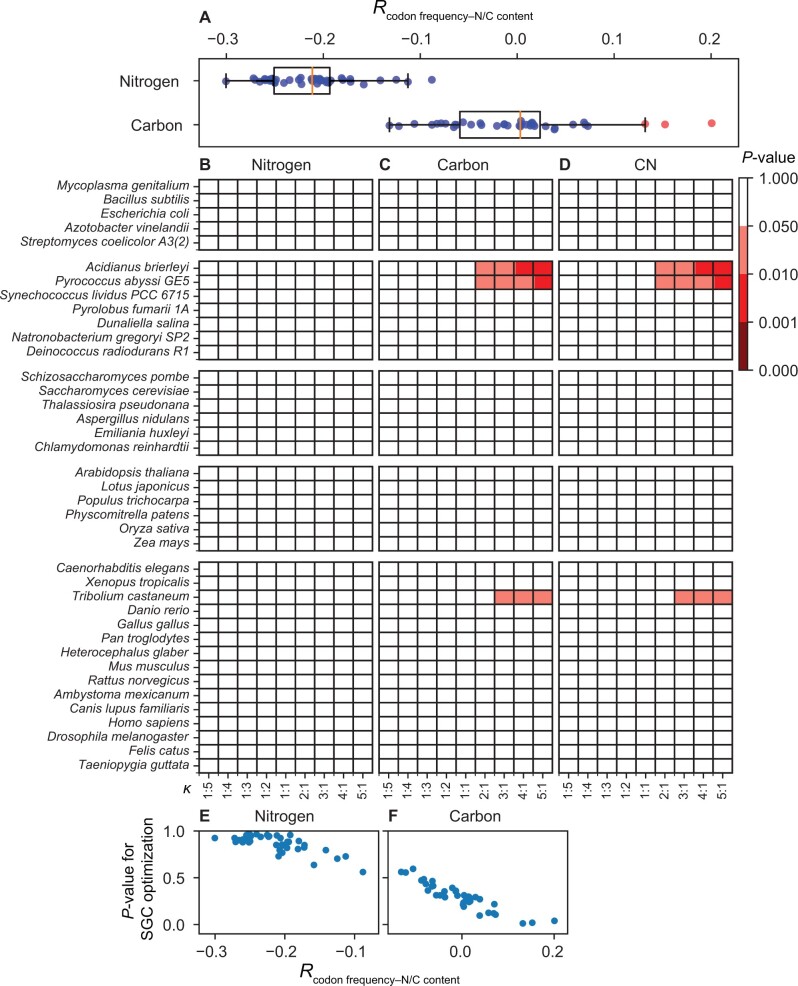
Testing the optimization of the SGC for resource conservation using RGCs generated by Shenhav and Zeevi’s method and nERMC. (*A*) Pearson’s correlation (*R*_codon frequency–N/C content_) between the genomic frequency of a codon and the number of nitrogen or carbon atoms in its encoded amino acid in each of 39 species examined. Each dot represents one species. A dot for nitrogen or carbon is marked in red if one or more of the nine examined *κ* (transition/transversion mutation rate ratio) values yield significant results in the corresponding species in (*B*) or (*C*); otherwise it is marked in blue. The box plot shows the distribution of the 39 data points, with the left and right edges of the box representing the first (*qu*_1_) and third (*qu*_3_) quartiles, respectively, the vertical line inside the box indicating the median (*md*), and the whiskers extending to the most extreme values inside inner fences, *md*±1.5(*qu*_3_−*qu*_1_). (*B*–*D*) Heat map of the significance level of the optimization of the SGC for conservation of nitrogen (*B*), carbon (*C*), or both carbon and nitrogen (*D*). Colors indicate the nominal *P* value, which is the fraction of RGCs whose nERMC is smaller than that of the SGC. (*E* and *F*) Relationship between *R*_codon frequency–N/C content_ and the significance level of the optimization of the SGC for nitrogen (*E*) or carbon (*F*) conservation. The significance level of optimization is determined under *κ *= 3 because *κ* is around 3 in most species (Zou and Zhang 2021). Pearson’s correlation between *R*_codon frequency–N/C content_ and the significance level of optimization is −0.75 (*P *<* *0.0001) in (*E*) and −0.92 (*P *<* *0.0001) in (*F*).

To verify these predictions, we compared the nERMC between the SGC and 1 million RGCs generated following Shenhav and Zeevi (2020), under the respective empirical codon frequencies of the 39 species at a series of *κ* values ranging from 0.2 to 5. Indeed, under no condition does the SGC exhibit a significantly lower nERMC for nitrogen when compared with the RGCs (fig. 2*B*). By contrast, the SGC shows a significantly lower nERMC for carbon (fig. 2*C*) and CN (carbon and nitrogen) (fig. 2*D*) in three species under a few *κ* values, although the significance levels are much weaker than originally reported (Shenhav and Zeevi 2020). As predicted, the SGC is generally less optimized than RGCs for nitrogen conservation (reflected by *P *>* *0.5 in all species in fig. 2*E*), and the three species exhibiting significant SGC carbon conservation have the strongest codon preferences for carbon-rich amino acids (red dots in fig. 2*A*). Furthermore, as illustrated in figure 1, our results quantitatively verify that the stronger the resource-driven selection on codon frequencies, the less optimized the SGC is (fig. 2*E* and *F*; supplementary fig. S1*B* and *C*, Supplementary Material online). Clearly, the “optimization” of the SGC for nutrient conservation (Shenhav and Zeevi 2020) is unrelated to the origin and evolution of the SGC but a side effect of codon usage in present-day gene sequences.

We also repeated the above analysis by considering both positive and negative mutational costs but followed Shenhav and Zeevi in treating mutations involving stop codons. The results (supplementary fig. S2, Supplementary Material online) are similar to those in figure 2*B*–*D*, except that the mutational cost is significantly lower in the SGC than RGCs for carbon and CN in more cases, indicating that Shenhav and Zeevi’s observations were largely but not entirely owing to their neglect of negative mutational costs and that mistreating mutations involving stop codons also contributed. The reason for the latter finding is simple. Because stop codons are selectively underrepresented in coding sequences, random mutations under the SGC tend to increase the number of stop codons. The same is not true under most RGCs because the stop codons under the SGC are no longer stop codons under most RGCs. Consequently, Shenhav and Zeevi’s consideration of zero resource consumption by stop codons tends to lower the mutational cost under SGC relative to that under RGCs.

### Our Results Are Robust to Different Classes of RGCs

When generating RGCs, Shenhav and Zeevi did not allow the number of codons for an amino acid to deviate from that in the SGC, departing from the common practice in testing optimizations of the SGC (Haig and Hurst 1991; Geyer and Madany Mamlouk 2018). To examine whether the results in figure 2 obtained under the RGCs generated using Shenhav and Zeevi’s method are robust, we generated another million RGCs using the commonly used method (see Materials and Methods); the number of codons for a given amino acid varies from one to six among these RGCs. We then compared these RGCs with the SGC in terms of nERMC. In none of the 39 species was the SGC significantly better than the RGCs in nitrogen conservation (supplementary fig. S3*A*, Supplementary Material online). And in only one species (*Pyrococcus abyssi*) under some *κ* values was the SGC significantly better than the RGCs in carbon (supplementary fig. S3*B*, Supplementary Material online) or CN (supplementary fig. S3*C*, Supplementary Material online) conservation. Again, the apparent carbon and CN conservation of the SGC in this species is likely a side effect of its preference for codons encoding carbon-rich amino acids (see the second red dot from the right in fig. 2*A*). Furthermore, the statistical significance here disappears if we correct for multiple testing (due to testing under multiple *κ* values in multiple species).

## Conclusion

As we have shown, Shenhav and Zeevi’s finding of optimization of the SGC for nitrogen/carbon conservation was an artifact of inappropriately calculating mutational effects; their results no longer hold when this problem is remedied. More importantly, we showed that lowering nERMC of the SGC is intrinsically coupled with raising the nutrient usage of the present-day proteome. Because selection on future nutrient usage is much weaker than that on the present-day nutrient usage, nutrient-driven selection for resource conservation cannot possibly lower nERMC. In other words, optimization of the SGC for resource conservation is theoretically untenable.

## Materials and Methods

### Calculation of nERMC

We used the same method as Shenhav and Zeevi’s, except that we considered all mutations that do not involve stop codons whereas they considered all mutations that increase the nutrient content. That is, $\mathrm{nERMC}=\frac{\sum_{i=1}^{61}\sum_{j=1}^{61}w_{ij}[n\left(j\right)-n\left(i\right)]}{\sum_{i=1}^{61}\sum_{j=1}^{61}w_{ij}}$, where *i* refers to the *i*th sense codon in the code table, *n*(*i*) is the number of nitrogen or carbon atoms in the amino acid encoded by codon *i*, and *w*_ij_ is the relative frequency of conversion from codon *i* to *j*, which equals the frequency of codon *i* when *i* and *j* differ by a transversion, the frequency of codon *i* multiplied by *κ* when *i* and *j* differ by a transition, and 0 otherwise. The codon frequency data of the 39 taxa were from a previous paper (Athey et al. 2017) and provided to us by Dr Zeevi. A series of *κ* values (1/5, 1/4, 1/3, 1/2, 1, 2, 3, 4, and 5) were considered.

### Random Genetic Codes

In figure 2, the RGCs were generated using Shenhav and Zeevi’s method. Specifically, let us first call every four codons in the SGC that differ only at the third codon position as a box. To create a RGC, we randomly shuffled the positions of the boxes in the SGC with the constraint that the sole stop codon in a box can reach one of the other two stop codons by exactly one transition. Consequently, the number of codons encoding any amino acid in any RGC is the same as that in the SGC.

In supplementary figure S3, Supplementary Material online, the RGCs were generated following the conventional method (Haig and Hurst 1991). Specifically, starting from the SGC, we kept the positions of the three stop codons unchanged and shuffled the amino acid labels among the 20 synonymous codon sets. As a result, the block structure of synonymous codons in the SGC is maintained but the number of codons encoding a given amino acid can vary among RGCs.

Given how little we know about the actual process of the origin of the SGC, it is unclear which of the above two ways of generating RGCs is more meaningful and whether there are other more meaningful ways than these two ways. Rozhonova and Payne (2021) reported that, when the original ERMC was used, consistent evidence for the optimization of the SGC in nitrogen conservation was found in only one of the ten different ways of RGC generation tried.

## Supplementary Material

Supplementary data are available at *Molecular Biology and Evolution* online.

## Supplementary Material

msab239_Supplementary_Data

## References

[msab239-B1] Archetti M. 2004. Codon usage bias and mutation constraints reduce the level of error minimization of the genetic code. J Mol Evol. 59(2):258–266. 15486699 10.1007/s00239-004-2620-0

[msab239-B2] Athey J , Alexaki A, Osipova E, Rostovtsev A, Santana-Quintero LV, Katneni U, Simonyan V, Kimchi-Sarfaty C. 2017. A new and updated resource for codon usage tables. BMC Bioinformatics 18(1):391. 28865429 10.1186/s12859-017-1793-7 PMC5581930

[msab239-B3] Baudouin-Cornu P , Surdin-Kerjan Y, Marliere P, Thomas D. 2001. Molecular evolution of protein atomic composition. Science 293(5528):297–300. 11452124 10.1126/science.1061052

[msab239-B4] Berube PM , Rasmussen A, Braakman R, Stepanauskas R, Chisholm SW. 2019. Emergence of trait variability through the lens of nitrogen assimilation in Prochlorococcus. Elife 8:e41043. 30706847 10.7554/eLife.41043 PMC6370341

[msab239-B5] Elser JJ , Acquisti C, Kumar S. 2011. Stoichiogenomics: the evolutionary ecology of macromolecular elemental composition. Trends Ecol Evol. 26(1):38–44. 21093095 10.1016/j.tree.2010.10.006 PMC3010507

[msab239-B6] Elser JJ , Fagan WF, Subramanian S, Kumar S. 2006. Signatures of ecological resource availability in the animal and plant proteomes. Mol Biol Evol. 23(10):1946–1951. 16870683 10.1093/molbev/msl068

[msab239-B7] Freeland SJ , Hurst LD. 1998. The genetic code is one in a million. J Mol Evol. 47(3):238–248. 9732450 10.1007/pl00006381

[msab239-B8] Geyer R , Madany Mamlouk A. 2018. On the efficiency of the genetic code after frameshift mutations. Peer J. 6:e4825. 29844977 10.7717/peerj.4825 PMC5967371

[msab239-B9] Goodarzi H , Nejad HA, Torabi N. 2004. On the optimality of the genetic code, with the consideration of termination codons. Biosystems 77(1–3):163–173. 15527955 10.1016/j.biosystems.2004.05.031

[msab239-B10] Grzymski JJ , Dussaq AM. 2012. The significance of nitrogen cost minimization in proteomes of marine microorganisms. ISME J. 6(1):71–80. 21697958 10.1038/ismej.2011.72 PMC3246230

[msab239-B11] Haig D , Hurst LD. 1991. A quantitative measure of error minimization in the genetic-code. J Mol Evol. 33(5):412–417. 1960738 10.1007/BF02103132

[msab239-B12] McEwan CEA , Gatherer D, McEwan NR. 1998. Nitrogen-fixing aerobic bacteria have higher genomic GC content than non-fixing species within the same genus. Hereditas 128(2):173–178. 9687237 10.1111/j.1601-5223.1998.00173.x

[msab239-B13] Mende DR , Bryant JA, Aylward FO, Eppley JM, Nielsen T, Karl DM, DeLong EF. 2017. Environmental drivers of a microbial genomic transition zone in the ocean’s interior. Nat Microbiol. 2(10):1367–1373. 28808230 10.1038/s41564-017-0008-3

[msab239-B14] Rozhonova H , Payne JL. Forthcoming 2021. Little evidence the standard genetic code is optimized for resource conservation. *Mol Biol Evol.* 38(11):5127–5133. 10.1093/molbev/msab236 PMC8557452 34373928

[msab239-B15] Shenhav L , Zeevi D. 2020. Resource conservation manifests in the genetic code. Science 370(6517):683–687. 33154134 10.1126/science.aaz9642

[msab239-B16] Xu H , Zhang J. Forthcoming 2021. On the origin of frameshift-robustness of the standard genetic code. *Mol Biol Evol*. doi: 10.1093/molbev/msab164. 10.1093/molbev/msab164 PMC8476161 34043802

[msab239-B17] Zou Z , Zhang J. 2021. Are nonsynonymous transversions generally more deleterious than nonsynonymous transitions? Mol Biol Evol. 38(1):181–191. 32805043 10.1093/molbev/msaa200 PMC7783172

