# Supplementary material for: Is the Genetic Code Optimized for Resource Conservation?
Source: Mol Biol Evol. 2021 Aug 12;38(11):5122–6. doi: 10.1093/molbev/msab239 (PMC8557414; doi:10.1093/molbev/msab239)
Supplement: msab239_Supplementary_Data [file msab239_supplementary_data.zip › Supplementary Materials.pdf]

Supplementary Materials for
"Is the genetic code optimized for resource conservation?" by H. Xu & J. Zhang

The supplementary materials include:
Legends of supplementary figures
Figures S1-S3
Data S1. Transcriptomic codon frequencies in 6 species (in a separate Excel file)

Legends of supplementary figures

Fig. S1. Testing the optimization of the SGC for resource conservation using RGCs generated by Shenhav and Zeevi's method and nERMC. Transcriptomic instead of genomic codon frequencies are considered here. The six species used are listed in Data S1. (A) Pearson's correlation ($R_{\text{codon frequency–N/C content}}$) between the frequency of a codon and the number of nitrogen or carbon atoms in its encoded amino acid. Each dot represents a species and the data are presented in a box plot. (B-C) Relationship between $R_{\text{codon frequency–N/C content}}$ and the significance level of the optimization of the SGC for nitrogen (B) or carbon (C) conservation. The significance level of optimization is determined under $\kappa = 3$ because κ is around 3 in most species (Zou and Zhang 2021). Pearson's correlation and associated *P*-value are presented.

Fig. S2. Heat map of the significance level of the optimization of the SGC for conservation of nitrogen (B), carbon (C), or CN (both carbon and nitrogen) (D). Colors indicate the nominal *P* value, which is the fraction of RGCs whose nERMC is smaller than that of the SGC. The analysis here follows that in Fig. 2B-D except that mutations involving stop codons are treated as in Shenhav and Zeevi (2020).

Fig. S3. Testing the optimization of the SGC for resource conservation using nERMC and RGCs generated by the conventional method (see Materials and Methods). Heat map of the significance level of the optimization of the SGC for conservation of nitrogen (A), carbon (B), or both carbon and nitrogen (C). Colors indicate the nominal *P* value, which is the fraction of RGCs whose nERMC is smaller than that of the SGC.

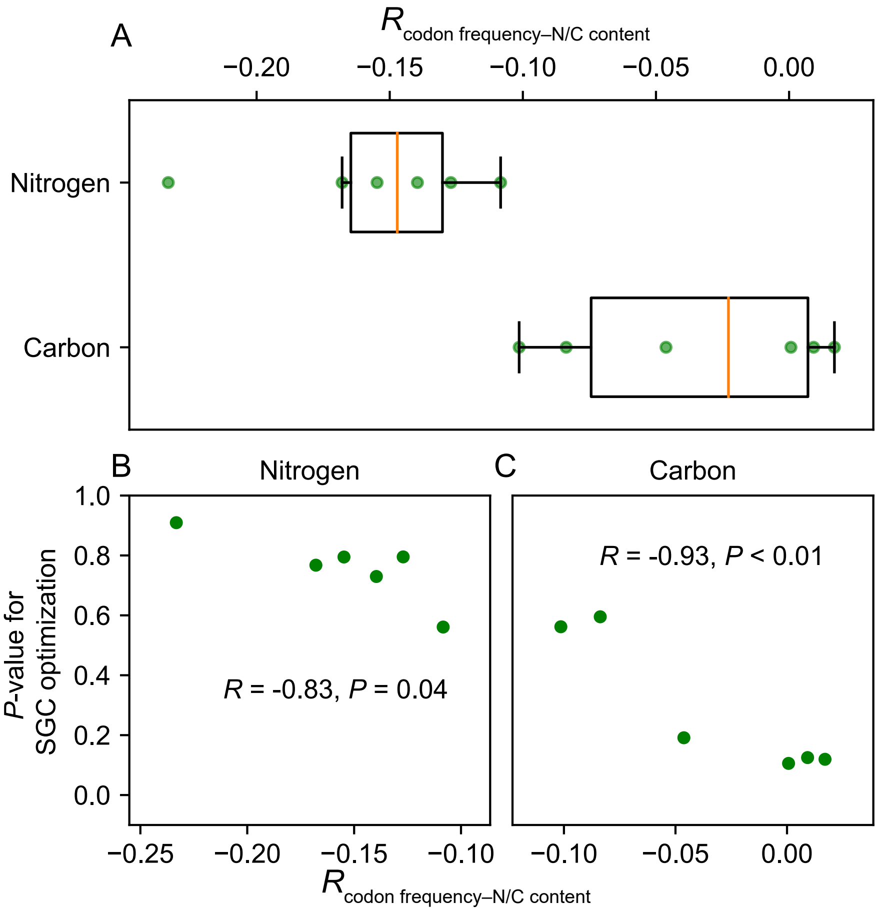
A
$R_{\text{codon frequency–N/C content}}$
−0.20
−0.15
−0.10
−0.05
0.00
Nitrogen
Carbon
B
Nitrogen
C
Carbon
1.0
0.8
0.6
0.4
0.2
0.0
P-value for SGC optimization
R = -0.83, P = 0.04
R = -0.93, P < 0.01
−0.25
−0.20
−0.15
−0.10
−0.10
−0.05
0.00
$R_{\text{codon frequency–N/C content}}$

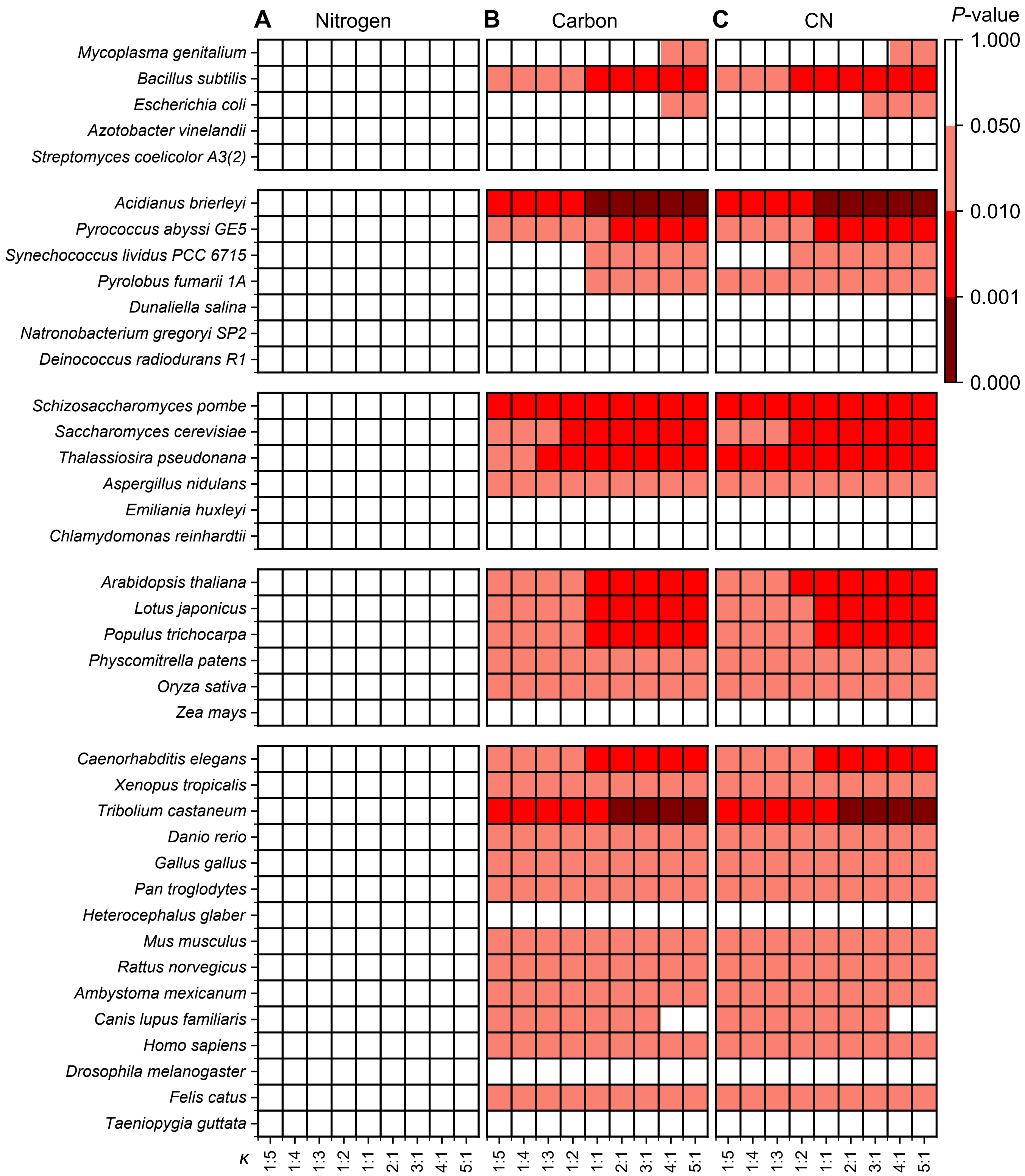

A
Nitrogen
B
Carbon
C
CN
P-value
1.000
0.050
0.010
0.001
0.000
Mycoplasma genitalium
Bacillus subtilis
Escherichia coli
Azotobacter vinelandii
Streptomyces coelicolor A3(2)
Acidianus brierleyi
Pyrococcus abyssi GE5
Synechococcus lividus PCC 6715
Pyrolobus fumarii 1A
Dunaliella salina
Natronobacterium gregoryi SP2
Deinococcus radiodurans R1
Schizosaccharomyces pombe
Saccharomyces cerevisiae
Thalassiosira pseudonana
Aspergillus nidulans
Emiliania huxleyi
Chlamydomonas reinhardtii
Arabidopsis thaliana
Lotus japonicus
Populus trichocarpa
Physcomitrella patens
Oryza sativa
Zea mays
Caenorhabditis elegans
Xenopus tropicalis
Tribolium castaneum
Danio rerio
Gallus gallus
Pan troglodytes
Heterocephalus glaber
Mus musculus
Rattus norvegicus
Ambystoma mexicanum
Canis lupus familiaris
Homo sapiens
Drosophila melanogaster
Felis catus
Taeniopygia guttata
K
1:5
1:4
1:3
1:2
1:1
2:1
3:1
4:1
5:1

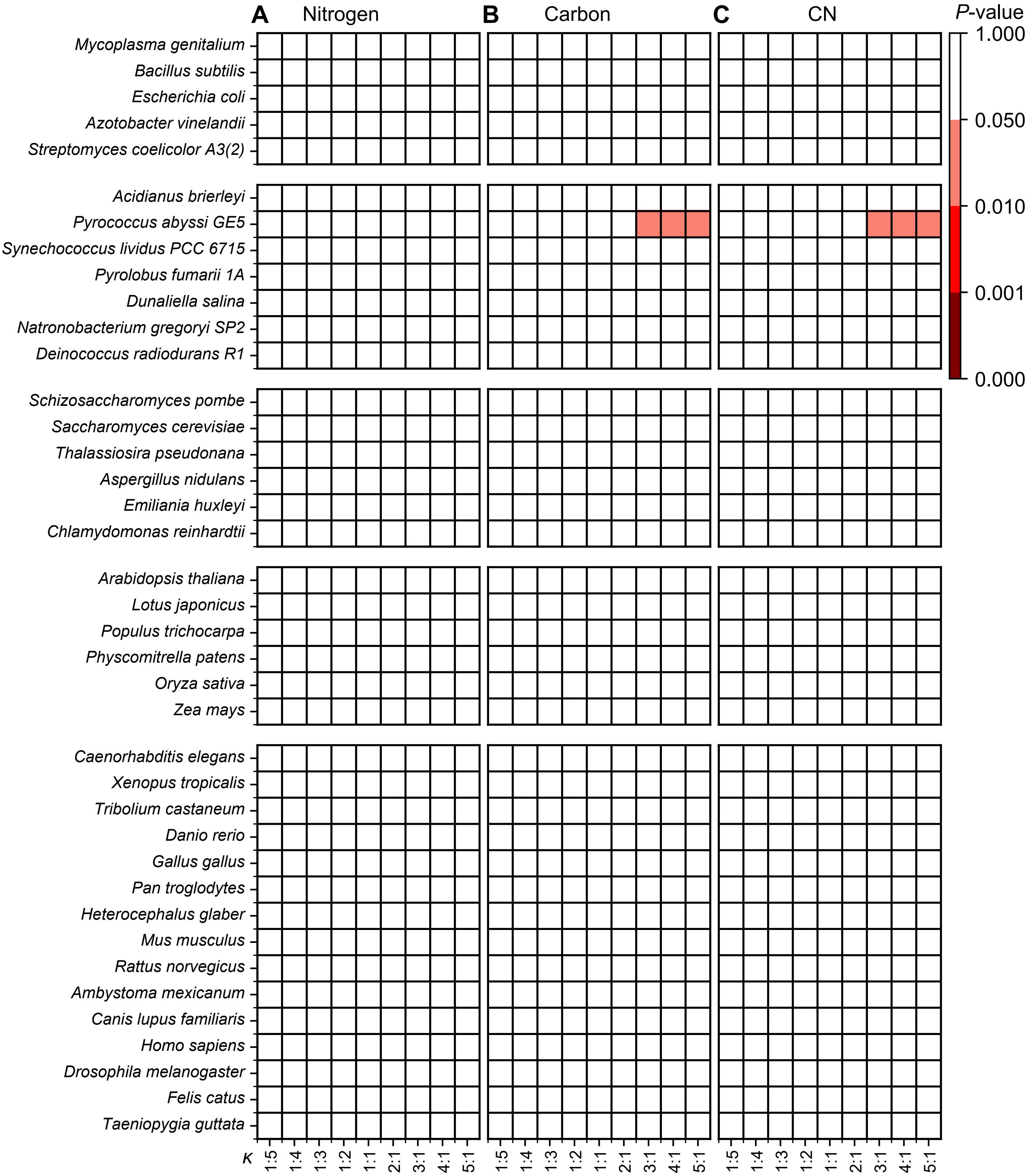
A
Nitrogen
B
Carbon
C
CN
P-value
1.000
0.050
0.010
0.001
0.000
Mycoplasma genitalium
Bacillus subtilis
Escherichia coli
Azotobacter vinelandii
Streptomyces coelicolor A3(2)
Acidianus brierleyi
Pyrococcus abyssi GE5
Synechococcus lividus PCC 6715
Pyrolobus fumarii 1A
Dunaliella salina
Natronobacterium gregoryi SP2
Deinococcus radiodurans R1
Schizosaccharomyces pombe
Saccharomyces cerevisiae
Thalassiosira pseudonana
Aspergillus nidulans
Emiliania huxleyi
Chlamydomonas reinhardtii
Arabidopsis thaliana
Lotus japonicus
Populus trichocarpa
Physcomitrella patens
Oryza sativa
Zea mays
Caenorhabditis elegans
Xenopus tropicalis
Tribolium castaneum
Danio rerio
Gallus gallus
Pan troglodytes
Heterocephalus glaber
Mus musculus
Rattus norvegicus
Ambystoma mexicanum
Canis lupus familiaris
Homo sapiens
Drosophila melanogaster
Felis catus
Taeniopygia guttata
K
1:5
1:4
1:3
1:2
1:1
2:1
3:1
4:1
5:1
